# Impacts of warming on phytoplankton abundance and phenology in a typical tropical marine ecosystem

**DOI:** 10.1038/s41598-018-20560-5

**Published:** 2018-02-02

**Authors:** John A. Gittings, Dionysios E. Raitsos, George Krokos, Ibrahim Hoteit

**Affiliations:** 1 Department of Earth Science and Engineering, King Abdullah University of Science and Technology (KAUST), Thuwal, 23955-6900 Kingdom of Saudi Arabia; 2 Remote Sensing Group, Plymouth Marine Laboratory, Prospect Place, The Hoe, PL1 3DH United Kingdom

## Abstract

In the tropics, thermal stratification (during warm conditions) may contribute to a shallowing of the mixed layer above the nutricline and a reduction in the transfer of nutrients to the surface lit-layer, ultimately limiting phytoplankton growth. Using remotely sensed observations and modelled datasets, we study such linkages in the northern Red Sea (NRS) - a typical tropical marine ecosystem. We assess the interannual variability (1998–2015) of both phytoplankton biomass and phenological indices (timing of bloom initiation, duration and termination) in relation to regional warming. We demonstrate that warmer conditions in the NRS are associated with substantially weaker winter phytoplankton blooms, which initiate later, terminate earlier and are shorter in their overall duration (~ 4 weeks). These alterations are directly linked with the strength of atmospheric forcing (air-sea heat fluxes) and vertical stratification (mixed layer depth [MLD]). The interannual variability of sea surface temperature (SST) is found to be a good indicator of phytoplankton abundance, but appears to be less important for predicting bloom timing. These findings suggest that future climate warming scenarios may have a two-fold impact on phytoplankton growth in tropical marine ecosystems: 1) a reduction in phytoplankton abundance and 2) alterations in the timing of seasonal phytoplankton blooms.

## Introduction

**Figure 1:**
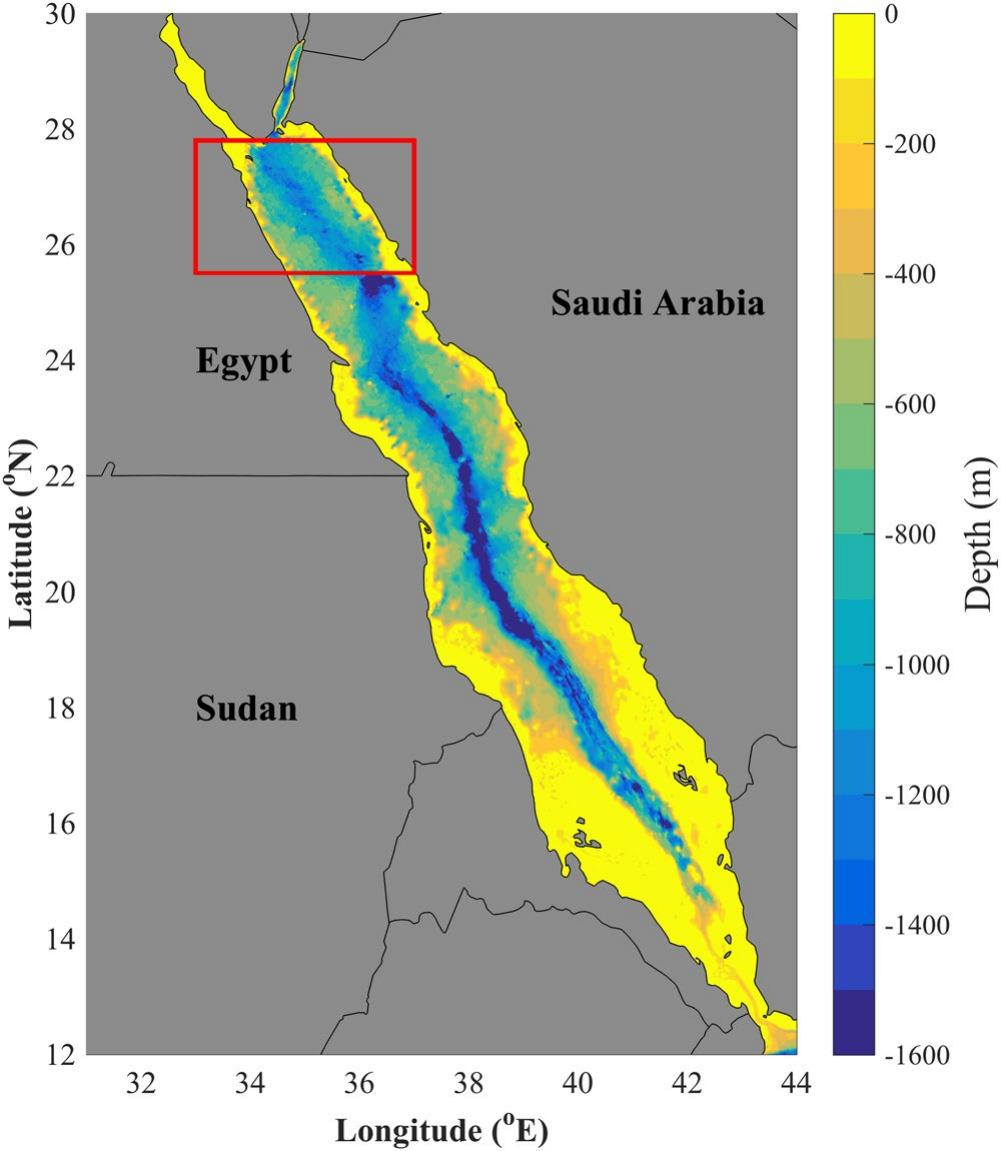
Map displaying the bathymetry of the Red Sea and geographical location of the northern Red Sea province (shown by red box). Figure 1 was produced using the software package MATLAB (version R2015b, https://www.mathworks.com).

Tropical regions harbour some of Earth’s most productive and diverse marine ecosystems, which provide important services for human populations^1^. The Red Sea (Fig. 1), the world’s northernmost tropical sea, is an important economic asset (via tourism, shipping and fisheries)^2–4^ and hosts one of the longest coral reef systems on Earth, which supports high levels of biodiversity and endemism^5^. Evidence indicates that the Red Sea, which has formerly been classified as a fast-warming Large Marine Ecosystem (LME)^6^, underwent an abrupt, step-wise temperature increase in response to global warming trends, which began in the mid-90s and has persisted until the present day^7^.

In tropical marine ecosystems, warmer conditions may reduce the abundance and primary productivity of phytoplankton - microscopic photosynthetic algae that form the base of the marine food web. This decrease results from enhanced stratification, less vertical mixing and reduced nutrient supply to the euphotic zone^8,9^. Despite this, warmer climatic conditions (i.e., positive phases of the El Niño Southern Oscillation - ENSO) have been linked with higher biomass over a large region of the Red Sea, due to increased (wind-induced) horizontal nutrient transport from the Indian Ocean^10^. The northern Red Sea (NRS, red box in Fig. 1) is unique in the fact that it is the only region in the Red Sea that does not emulate this pattern. Instead, phytoplankton dynamics in the NRS follow a typical tropical regime, where warmer, stratified conditions contribute to less vertical mixing and a reduction in phytoplankton abundance^10^.

The NRS is characterised by a distinct winter phytoplankton bloom that occurs when colder atmospheric conditions contribute to significant heat loss over the region, and convective mixing (overturning) transports nutrients from deeper waters into the surface layers^11–17^. The NRS winter bloom is important for the regional ecosystem. For instance, the seasonal increase in abundance may be paramount for zooplankton dynamics (e.g. feeding and maturation), and the reproductive strategies of reef organisms (e.g. molluscs and fish), as already highlighted in the Gulf of Aqaba (the northernmost extension of the NRS)^18–20^.

Phytoplankton abundance and phenology (bloom timing) can be categorised as ‘ecological indicators’ that can be used to assess the condition of the pelagic ecosystem^21–23^. Interannual fluctuations in the timing of phytoplankton growth can have far-reaching ecosystem impacts, as the fitness and recruitment of organisms at higher trophic levels is ultimately dependent on their temporal synchrony with food availability (match – mismatch hypothesis^24,25^). Previous studies for different oceanic regions have revealed that changes in phytoplankton phenology can negatively impact the survival of commercially important species^22,26^.

Due to the lack of adequate long-term, *in situ* biological datasets, the NRS is relatively unexplored in the context of large-scale phytoplankton dynamics at an interannual level. One alternative that can be utilised to conduct interannual analyses is the use of satellite-derived chlorophyll-a (Chl-a, an index of phytoplankton biomass) datasets^27^, which provide valuable information about phytoplankton dynamics over long time periods. The recent development of the Ocean Colour Climate Change Initiative (OC-CCI) project by the European Space Agency (ESA)^28^ [http://www.esa-oceancolour-cci.org] led to the conception of a high quality, global-scale, error-characterised Chl-a time-series, generated by merging datasets from multiple ocean-colour sensors. Currently, the OC-CCI dataset is one of the longest (~18 years) and most consistent time-series of Chl-a available, and its use may therefore be suitable for assessing the influence of climate-driven alterations on phytoplankton.

In this study, we centre our investigation on elucidating the potential response of phytoplankton dynamics in the NRS to regional warming. We use the OC-CCI dataset to assess the long-term interannual variability of both phytoplankton abundance (as indexed by total satellite-derived Chl-a concentration) and phenological indices (timing of bloom initiation, duration and termination). The mechanistic links between the observed variability in phytoplankton dynamics and regional abiotic factors (SST, mixed layer depth [MLD] and air-sea heat fluxes) are also explored.

## Results

### Temporal patterns and links between chlorophyll-a and SST

**Figure 2:**
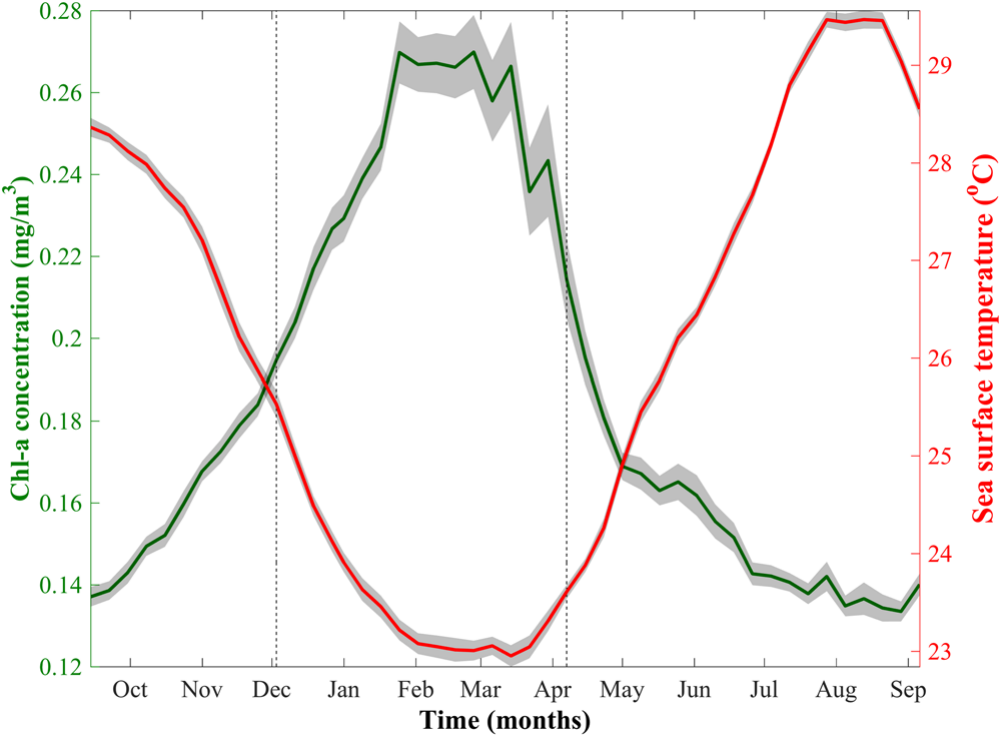
Seasonal climatology of satellite-derived Chl-a and sea surface temperature (based on 8-day composites) in the northern Red Sea averaged over the period 1998–2015. Grey shading represents +/− one standard error for both parameters. The grey-dashed vertical lines represent the average timings of phytoplankton bloom initiation and termination for the period 1998–2015.

Based on 18 years of satellite-derived Chl-a, we computed the seasonal climatology of phytoplankton biomass and phenology (Fig. 2, see methodology). The NRS phytoplankton bloom initiates at the beginning of December, terminates in early April and has a mean duration of ~4 months. Highest Chl-a concentrations are detected at the end of January and subsist until mid-March, representing the general peak of the bloom. During this period, Chl-a remains high and is generally stable. The seasonal climatology of Chl-a is a near-perfect anti-correlation (n = 46, ρ = −0.97, p < 0.000001) of the SST seasonal cycle (red line, Fig. 2). Lowest SST coincides with the peak of the bloom and begins to increase at the end of March. Maximum SST occurs from mid-July to mid-September, alongside the occurrence of minimum Chl-a concentrations.

**Figure 3:**
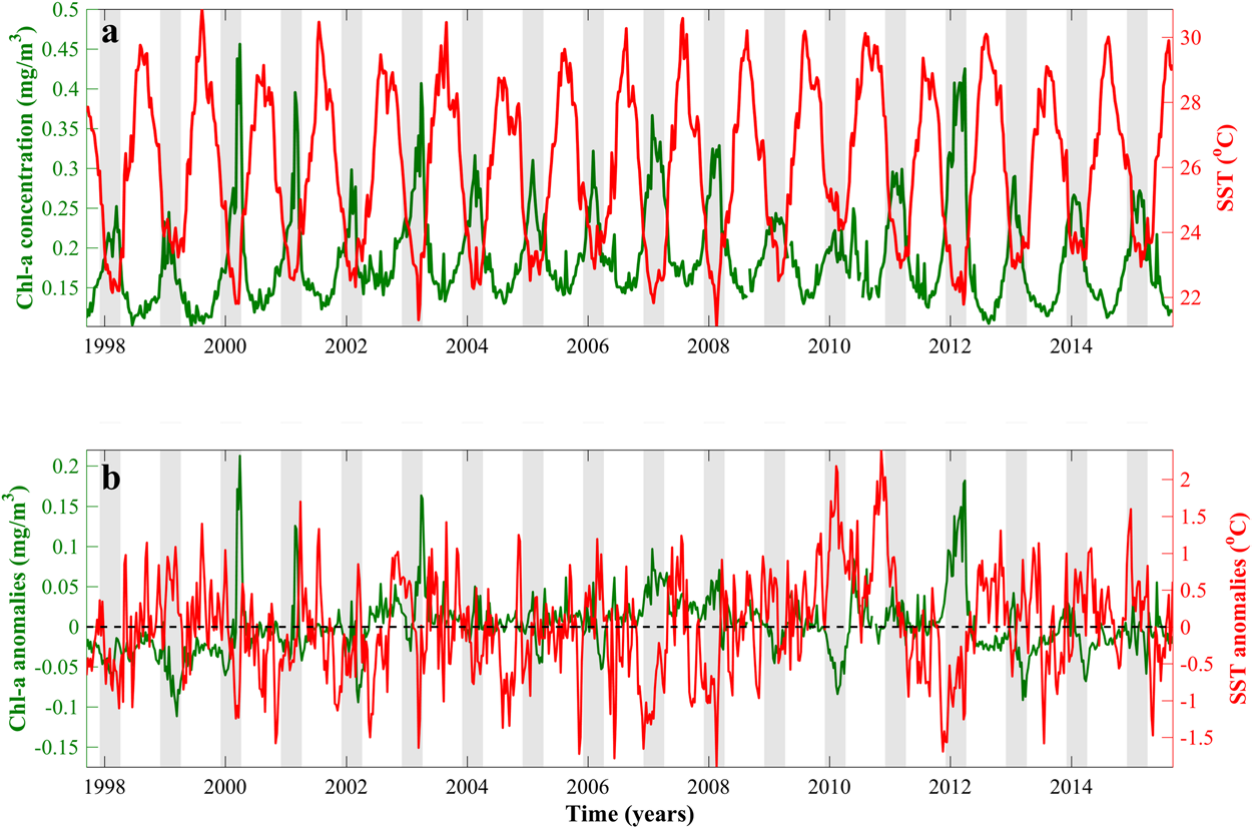
(**a**) Time series of satellite-derived Chl-a concentration and sea surface temperature (8-day averages) for the northern Red Sea (1998–2015). (**b**) Time series of corresponding satellite-derived Chl-a and SST anomalies (observed value minus overall mean). Grey-shaded vertical bars represent the bloom period (early- December – early-April) defined by the phenology analysis (Fig. 2).

The interannual variability of Chl-a is dominated by the seasonal cycle and highest values consistently occur during the bloom period defined by the phenology analysis (early-December to early-April, grey-shaded vertical bars, Fig. 3a). To remove the seasonality of the time series and clearly isolate interannual events, we produced the respective anomalies of Chl-a and SST (Fig. 3b). Substantially reduced winter Chl-a concentrations can be observed in 1999 and 2010, which co-occur with warmer winter SSTs (a similar response to elevated winter SST anomalies is also evident in 2006, 2013 and 2014, although to a weaker extent, Fig. 3a,b). Oppositely, higher Chl-a concentrations (alongside considerably colder SSTs) can be identified in 2007, 2008 and 2012, and appear to remain higher over the whole bloom period (Fig. 3). Notably higher Chl-a anomalies occur towards the end of the bloom period in 2000, 2001 and 2003 (Fig. 3b), although these events are generally short-lived (2–3 weeks) and the corresponding SST anomalies are more variable.

**Figure 4:**
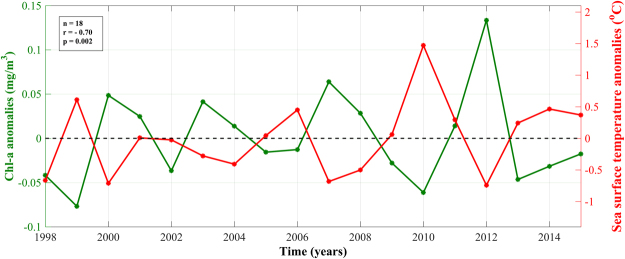
Time series of satellite-derived Chl-a and sea surface temperature anomalies (observed value minus overall mean) averaged over the general peak of the phytoplankton bloom period (late-January – late-March, Fig. 2).

To emphasize the interannual pattern of Chl-a and its relationship with SST, we averaged both parameters during the general peak of the bloom (late-January – mid-March, Fig. 4). Several anomalously warm winter peaks can be observed in the time series, namely occurring in 1999 and 2010 and to a lesser extent in 2006 and 2014. Generally, these years are characterised by some of the weakest winter blooms over the last 18-years. Conversely, 2000, 2007 and 2012 (the coldest winter periods) co-occur with the most intense bloom peaks. Overall, a clear, significant, negative relationship between Chl-a and SST can be observed at both seasonal (Fig. 2) and interannual (Figs 3 and 4) scales.

### Spatial patterns of phytoplankton phenology under warmer scenarios

**Figure 5:**
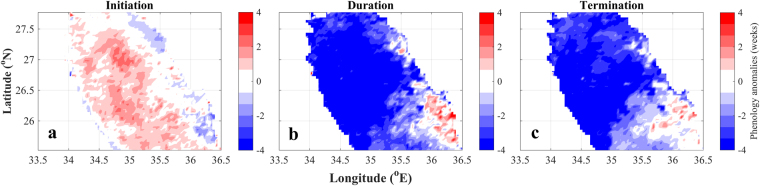
Maps of the northern Red Sea displaying differences between phytoplankton phenology indices generated for ‘warm’ (1999, 2006, 2010, 2011, 2013, 2014, 2015) and cold (1998, 2000, 2003, 2004, 2007, 2008, 2012) years of the study period (based on the analysis presented in Fig. 4). These maps are representative of the phenology response during ‘warm’ climate conditions over the northern Red Sea region. During warmer periods, the initiation of phytoplankton growth is delayed by ~1–4 weeks and terminates up to 4 weeks earlier, ultimately leading to a shorter bloom duration overall. Figure 5 was produced using the software package MATLAB (version R2015b, https://www.mathworks.com).

To investigate the response of the ecosystem to ‘warmer’ conditions in the NRS, we created spatial composites of phenology indices (initiation, duration and termination) by averaging all of the warmer/colder years of the study period (based on Fig. 4). Years when SST was not evidently above/below average (e.g. 2001, 2002, 2005 and 2009) were not used to generate the composite images. The resultant maps (Fig. 5) display the differences between these composites and are representative of the response of phytoplankton phenology to warmer scenarios. Under warmer conditions, bloom initiation across most of the NRS occurs later (~1–4 week delay). Both bloom duration and termination exhibit a similar, and perhaps more striking response during warmer phases; termination and duration are, on average, 4 weeks earlier/shorter respectively across the majority of NRS (Fig. 5b,c). The exception to this is the south/southeast region of the NRS, where the response varies from no change to longer bloom duration and delayed termination (~2–3 weeks). No change in bloom initiation can be identified in the northeast region of the NRS and to a lesser extent in the southeast.

### Physical controls on phytoplankton abundance and phenology

**Figure 6:**
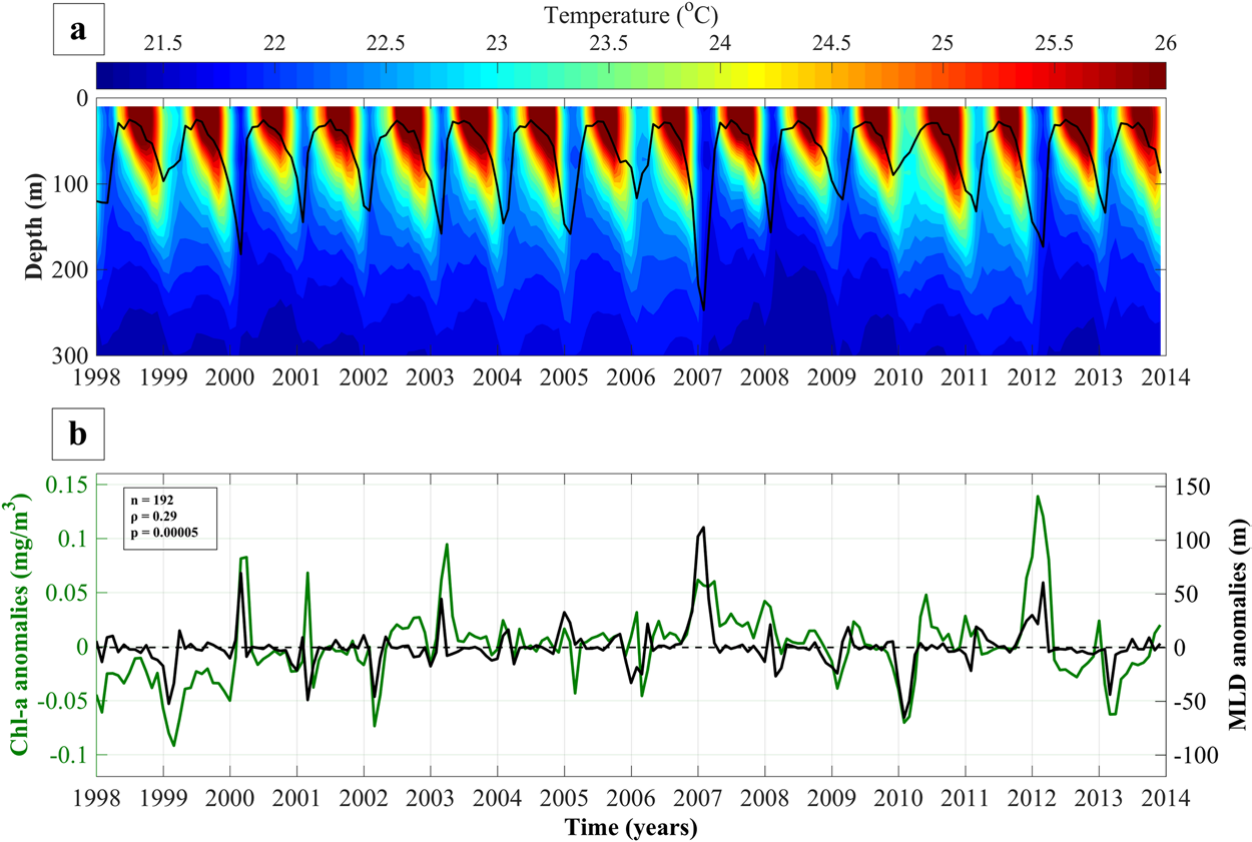
(**a**) Contour plot displaying vertical temperature profiles and mixed layer depth (MLD) in the northern Red Sea for the period 1998–2014. (**b**) Monthly anomalies of satellite-derived Chl-a concentrations and modelled mixed layer depth anomalies for the equivalent period. Positive MLD anomalies represent deeper mixing conditions.

We have highlighted the interannual oscillations in phytoplankton biomass, corresponding links with SST and the identification of distinct warm and cold years. Furthermore, we have assessed the impact of warmer conditions on phytoplankton bloom timing. In order to further develop these findings in a physical context, we examined the relationships between Chl-a concentration, vertical temperature profiles (based on outputs acquired from the MIT general circulation model - see methodology) and the corresponding MLD (Fig. 6).

Anomalously warm SSTs during the winter bloom peaks of 1999, 2006 and 2010 (Fig. 4) are accompanied by similarly elevated temperatures (~23.5–24 °C) in the upper 100 metres of the water column and shallower MLD (80–120 metres, solid black line, Fig. 6a). In contrast, cooler temperatures (~21–21.6 °C) in 2000, 2007 and 2012 parallel a substantial deepening of the MLD (~180–250 metres, Fig. 6a). The Chl-a response to variations in the MLD is apparent, as represented by the moderately strong, positive correlation (n = 192, ρ = 0.29, p = 0.00005) between monthly Chl-a and MLD anomalies (Fig. 6b).

**Figure 7:**
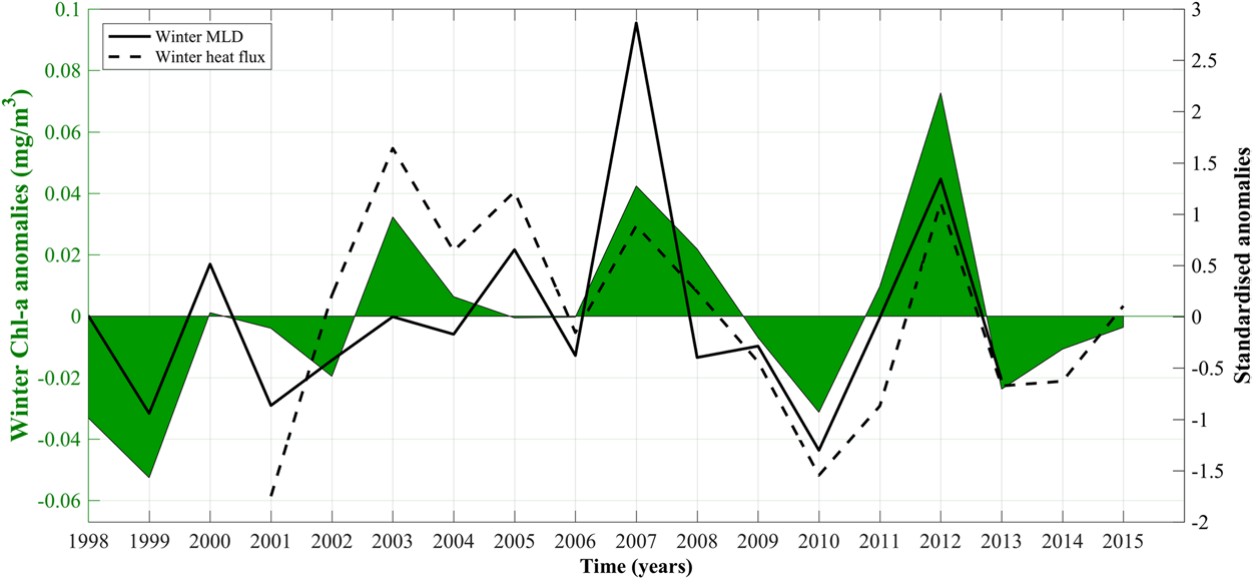
Winter (October – April) satellite-derived Chl-a anomalies (left y-axis) plotted against standardised winter anomalies of mixed layer depth and heat flux (right y-axis). Physical variables are presented as standardised anomalies for the purpose of plotting them on a single axis.

To investigate whether the interannual variability of Chl-a concentration is related to density changes driven by warming/cooling of the surface layers, we examined the relationships between the average winter (October – April) anomalies of air-sea heat-fluxes, MLD and Chl-a in the NRS (Fig. 7). This period was chosen in order to encapsulate the period of preconditioning in early winter when vertical mixing may have only just started to occur. Negative heat-flux anomalies are significantly correlated with a shallower MLD (n = 13, ρ = 0.77, p = 0.003) and lower Chl-a anomalies (n = 15, ρ = 0.66, p = 0.009), whilst a shallower MLD is significantly associated with decreased winter Chl-a concentrations (n = 16, ρ = 0.64, p = 0.009).

**Figure 8:**
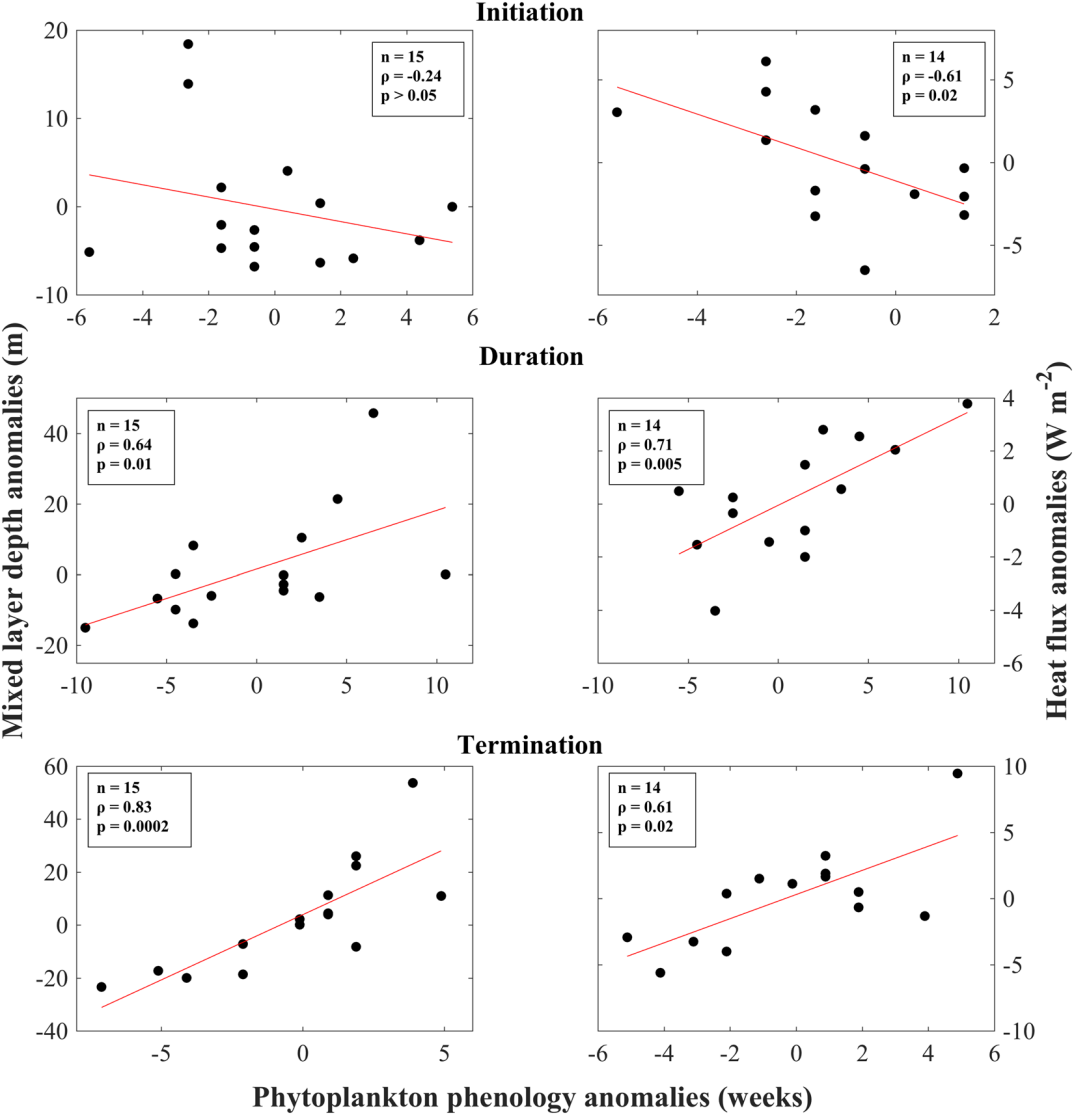
Scatterplots of spatially averaged mixed layer depth and heat flux anomalies vs. annual anomalies of phytoplankton phenology metrics i.e., bloom initiation, duration and termination. The red lines represent the linear regression between the two variables. Mixed layer depth and heat flux anomalies were averaged over periods corresponding to each phenology metric (*initiation*: October – December, *duration*: October - April, *termination*: February - April). The mixed layer depth and heat flux data were available for the periods 1998–2013 and 2001–2015 respectively.

Based on the response of phytoplankton phenology to warmer conditions (Fig. 5), we explored further the potential influence of physical variables in controlling phytoplankton bloom timing. Anomalies of MLD and heat flux (averaged over the periods characterising each phenological index [initiation: October – December, duration: October – April, termination: February – April]) were plotted against corresponding annual phenology anomalies (Fig. 8). Note that the threshold method^27,29^ was unable to detect the occurrence of clear bloom timings in 2010, and this year was thus excluded from the analysis. The reasons for this will be discussed in the following section. Results show a negative relationship between bloom initiation anomalies and both MLD/heat flux (although this is relatively weak and not significant for MLD, Fig. 8). Highly significant, positive relationships occur between phytoplankton bloom duration anomalies and MLD/heat flux (Fig. 8). Analogous results are also revealed by the relationships between termination anomalies and MLD/heat flux (Fig. 8). We note that relationships between phenological indices and SST were also tested (results not shown). SST is uncorrelated with bloom initiation and duration anomalies, but exhibits a strong, significant correlation with termination anomalies (n = 17, ρ = − 0.69, p = 0.002).

## Discussion

We examined the interannual variability of phytoplankton abundance and the timing of events for the first time in the NRS. Our results reveal that phytoplankton biomass is coupled with SST at seasonal and interannual timescales (Figs 2, 3 and 4), signifying that colder SSTs are a useful indicator of convection events that contribute to elevated nutrients in the surface layer and increased phytoplankton biomass.

Inclusively, the interannual variability of Chl-a concentrations is determined by the strength of vertical mixing in the water column (Figs 6b and 7). Recent studies in the NRS have highlighted the importance of air-sea heat exchanges in vertical mixing and the ventilation of the deep layers^30^. The variability of vertical mixing is largely related to density changes (through buoyancy forcing by net heat fluxes) that drive the variability of the mixed layer (Fig. 7). These changes are manifested as direct heat exchanges (i.e. cooling/warming of the surface layers) and indirectly through latent (evaporative) heat fluxes that increase salinity. Years characterised by reduced Chl-a (e.g. 1999 and 2010) coincide with elevated surface temperatures (~1–1.5 °C higher than average) and a remarkably shallower MLD following weaker air-sea heat fluxes (Figs 3, 4, 6 and 7). As nutrient concentrations generally increase with depth^31,32^, a relaxation of vertical mixing reduces nutrient supply to the euphotic zone, diminishing phytoplankton productivity. Inversely, stronger mixing events that penetrate deeper in the water column will replenish nutrients in the surface layer, inducing more intense phytoplankton blooms. These results are supported by Calbert *et al*.^33^ who demonstrated that bloom initiation in the central Red Sea likely occurs following MLD deepening and the entrainment of nutrients into the upper layer, allowing seed populations to flourish rapidly. In addition, Genin *et al*.^34^ reported that 96% of the interannual variability found in winter Chl-a concentration in the Gulf of Aqaba, over an 8-year period, could be explained by variations in air-sea heat fluxes, which control the maximum depth of mixing.

We note that other physical factors may contribute to the transport of nutrients to the surface layer and the subsequent stimulation of phytoplankton growth in the NRS. Rigorous eddy activity in the Red Sea^35,36^ and especially the presence of a distinct cyclonic gyre between 26°N and 27°N in the NRS has been reported in previous works^13,37,38^. Cyclonic activity is known to stimulate the upwelling of nutrients from deeper waters to the surface layer. The intensity of the cyclonic circulation depends on density gradients that are directly related to air sea exchanges. Thus, both mechanisms (convective mixing and cyclonic activity) are to some degree controlled by air-sea heat fluxes and may act to transport nutrients from deeper waters to the surface layer^39^. Papadopoulos *et al*.^15^ described how an increase in the NRS cyclonic gyre intensity leads to persistent upwelling, prolonged negative SST anomalies (colder temperatures), ultimately contributing to stronger phytoplankton blooms in the following winter when convective mixing occurs. These findings are consistent with our analysis and also explain why the strongest atmospheric forcing (e.g. in 2003, Fig. 7) may not always co-occur with the deepest MLD or the strongest phytoplankton blooms.

Maximum winter SSTs in 1999 and 2010 are associated with reduced heat exchanges with the atmosphere (heat flux data not available for 1999), the shallowest MLDs and lowest Chl-a concentrations throughout the time series (Figs 3, 4 and 7). These years may represent a delayed response to positive ENSO phases that occurred in the preceding years. The 1997/98 El Niño was one of the largest to occur in the 20^th^ century, whilst the 2009/10 El Niño was associated with record-breaking SSTs in the central Pacific Ocean^40,41^. Through ENSO teleconnections, global air temperatures may take up to six months to increase after an El Niño event^42,43^ and the warming response of the Red Sea to rising air temperatures has been shown to be lagged by approximately one month^7^. Thus, a ~7–8 month lag in the NRS SST response following an El Niño event is reasonable, and may explain the high temperatures and limited mixing observed in the winters of these years. Indeed, positive ENSO phases have been linked with increased stratification, a deepening of the nutricline and reduced Chl-a concentrations in other tropical regions such as the Equatorial Pacific and western Indian Oceans^8,44,45^. Due to the exceptionally high temperatures and limited mixing in 2010, we were unable to compute phenology metrics for the 2010 bloom. Visual analysis of the 2010 seasonal time series (figure not shown) confirmed that no discernible bloom could be identified during this year. It is worth noting that the NRS may be affected by other climatic modes. For instance, Papadopoulos *et al*.^46^ demonstrated that extreme winter heat loss events over the NRS are associated with the eastern Mediterranean lower-atmospheric circulation, which in turn may be partially controlled by the North Atlantic Oscillation (NAO). However, further investigation is required in order to fully elucidate the interactions between different climate modes and heat exchanges over the NRS.

The observed timings of the bloom peak (late-January - mid-March) and termination (early-April, Fig. 2) are consistent with the findings of Racault *et al*.^29^, who computed phenological indices for a small region in the central NRS. However, our evaluation of the average bloom initiation (early December) is later in comparison to the aforementioned study, which indicated that initiation occurs in November. The discrepancy between the two studies can probably be explained by differences in the threshold criteria used for the computation of the phenological indices. The use of a higher threshold criterion in our study (15% instead of the 5% threshold utilised in Racault *et al*.^29^, Siegel *et al*.^47^ and other literature, see methodology) likely resulted in a more delayed initiation estimate, as more time is required for Chl-a concentrations to exceed the specified threshold value.

Over the last 40 years, the global oceans have warmed at a rate of ~0.1 °C per decade in the upper water column^48^ and increasing Northern Hemisphere temperatures have been directly linked with the abrupt warming of the Red Sea^7^. Our results signify that phytoplankton phenology in the NRS is altered under such warmer climate scenarios. A delayed initiation under warm conditions (~1–4 weeks, Fig. 5a) is likely related to the time it takes for required buoyancy forcing to deepen the mixed layer to depths where nutrients are abundant enough to sustain phytoplankton growth. Warmer winters, characterised by weak atmospheric forcing, will increase the time required for sea-surface cooling to drive mixing at sufficient depths and re-distribute nutrients to the surface. Contrariwise, during cooler winters, increased air-sea heat fluxes and colder temperatures are likely to subsist at the commencement of the winter period, contributing to an earlier induction of convective mixing and an earlier phytoplankton bloom. Our analysis supports this theory, as bloom initiation anomalies are negatively correlated with the interannual variability of atmospheric forcing (i.e. weaker heat flux corresponds to later bloom initiation, Fig. 8). The relationship between phenology and heat flux/MLD is also evident in the termination of the bloom: warm (cold) winters associated with reduced (elevated) heat fluxes and shallower (deeper) MLDs are significantly linked to phytoplankton blooms that terminate earlier (later, Fig. 8). Weak vertical mixing during warmer winters contributes to low nutrient concentrations that will be quickly consumed by phytoplankton, thus restricting the period of phytoplankton growth. Furthermore, the onset of re-stratification and the shallowing of the MLD will occur more rapidly under warmer conditions, inhibiting the vertical transport of nutrients into the surface layer earlier. In consideration of this, a shortening of the bloom duration in response to warming is a logical consequence of shifts in the timing of initiation and termination, which themselves, are ultimately reliant on the inception of winter mixing and the onset of post-winter re-stratification respectively. This is comparable with the global analysis of Racault *et al*.^27^, who found that the duration of phytoplankton growth in the subtropical gyres of the Southern Hemisphere is dependent on both the timing of bloom initiation and termination.

Despite the clear negative trend, the lack of any significant relationship between MLD and bloom initiation anomalies (Fig. 8) may be attributed to the possibility that MLD dynamics are highly spatially heterogeneous. The commencement of convection and mixing are usually localised events limited to specific areas and both enable the vertical redistribution of nutrients. Thus, the strength of our statistical relationships could be affected by the spatial averaging of the MLD over the entire NRS, masking local convection events. Furthermore, the intrusion of water masses from the southeast may locally affect the MLD, also impacting spatially averaged values.

Although SST is a good indicator of overall phytoplankton biomass (Figs 2, 3 and 4), our results suggest that it is not as representative of changes in bloom timing in the NRS. For instance, SST anomalies only exhibited a strong, negative relationship with anomalies of bloom termination (results not presented). While the reasons for this are yet to be fully understood, we speculate that fluctuations in SST during the termination period (February – April) strongly reflect the onset of re-stratification during spring, which inhibits the re-distribution of nutrients to the surface layer. This may contrast to the initiation period (October – December), when SST anomalies are not able to capture the preconditioning phase of vertical mixing, which has not yet reached the surface layers. The lack of a relationship between SST anomalies and bloom duration could be explained by the fact that SST is more variable in comparison to heat fluxes or MLD. For example, SST has been shown to also depend on the general cyclonic circulation in the NRS^15^, the vigorous and highly variable eddy activity^35,36^, and the lateral advection of water masses from neighbouring regions following the general Red Sea circulation^13,16^. Thus, changes in MLD due to atmospheric forcing may occur alongside SST conditions that are discrepant with expected scenarios. This has been observed in other oceanic regions, such as the North Atlantic subpolar gyre, where a deeper MLD co-occurred in response to positive phases of the NAO, despite the presence of warmer SSTs^49^.

The contrasting spatial response of bloom termination and duration to warmer conditions between the north and southeast NRS (Fig. 5b,c) is an interesting result of our study. This contrast may directly reflect the regional dynamics related to the general circulation of the NRS as a response to atmospheric forcing. Density differences related to the north-south gradient of buoyancy forcing drive a general cyclonic circulation in the NRS^13^. This leads to the intrusion of fresher water masses from the south, which are transported northward into the NRS via a prominent eastern boundary current^13,16^. This intrusion is evident in the study of Raitsos *et al*.^10^ (their Fig. 3c,d) who analysed the response of Red Sea Chl-a to the Arabian monsoon. Thus, in contrast to the north/northwest region that is influenced by the vertical transport of nutrients from deeper layers, phytoplankton phenology in the southeast NRS may be partially controlled by the intrusion of fresher water masses from the south. Similarly, there may be potential losses of nutrients and phytoplankton over the open boundary in the western NRS. As our computations of phenology indices are based on area-averaged Chl-a concentrations for the whole NRS, we acknowledge that the inclusion of the southeast NRS could impact the results of our analysis. Despite this, we capture the overall signal of phytoplankton abundance and phenology using area-averaged values, and the relationships between phenology and air-sea heat flux/MLD are fairly robust (Fig. 8).

Overall, during warmer conditions (which are characterised by lower heat fluxes and more stratified conditions), the NRS phytoplankton bloom initiates ~1–4 weeks later, is ~4 weeks shorter in duration, and terminates ~4 weeks earlier (Figs 5 and 8). The NRS has been reported to be the fastest warming region in the Red Sea and is warming approximately four times faster than the average global ocean warming rate^50^. In consideration of this, as well as a potential increase in the frequency of extreme El Niño events^51^, there is a possibility of a two-fold impact of warmer climate scenarios on phytoplankton dynamics in the NRS: 1) a decrease in overall phytoplankton abundance; and 2) changes in the timing of the seasonal phytoplankton bloom. In coral reef ecosystems, phytoplankton is a direct food source for sponges^52^, bi-valves^53^ and pelagic larvae^54^. Thus, a reduction in food availability (quantity and time) may have severe ramifications for higher trophic levels in NRS coral reef complexes. Elevated temperatures in other reef ecosystems have been related to a significant reduction in Chl-a concentrations. For instance, a study in French Polynesia revealed a 50% reduction in fish larval supply to the reef due to increased larval mortality^55^ associated with lower Chl-a concentrations. Analogous impacts may occur in the NRS coral reefs, potentially affecting the recruitment of reef organisms and regional biodiversity. The ecological impacts of alterations in phytoplankton phenology could be manifested in numerous ways. The survival of higher trophic levels may be detrimentally impacted due to the mismatch between the timing of food availability (phytoplankton) and the presence of planktonic larvae^56,57^. A difference of 2–3 weeks in bloom phenology may inhibit the survival of herbivorous zooplankton and fish in subtropical regions^58^. A resultant decline in the survival and recruitment of larvae may occur if the initiation or termination of the bloom begins to occur later or earlier respectively, and larval spawning continues to match the original timing of the bloom prior to warming^59^. In light of this, if phytoplankton phenology in the NRS is altered more drastically in conjunction with current climate change trends, the potential ensuing negative impacts on commercially important species may be detrimental for human populations that depend on coastal fisheries resources for their sustenance and economy.

## Methodology

### Study area

Geographical limits of the NRS were chosen based on the Red Sea biological provinces presented in Raitsos *et al*.^12^ and are defined as 33°E–37°E and 25.5°N–27.8°N (red box in Fig. 1). For the purpose of this study, the Gulfs of Aqaba and Suez were not included in the analysis as they are regionally controlled by different dynamics^60^.

### Bathymetry data

Gridded bathymetry data used for the generation of Fig. 1 were acquired from the General Bathymetric Chart of the Oceans (GEBCO_2014 Grid, version 20150318, http://www.gebco.net).

### Satellite ocean-colour data

Version 3.1 of the ESA OC-CCI product^28^ was used in this study. This product consists of merged and bias-corrected Chl-a data from the Sea-Viewing Wide Field-of-View Sensor (SeaWiFS), Moderate Resolution Imaging Spectroradiometer (MODIS), Medium Resolution Imaging Spectrometer (MERIS) and Visible Infrared Imaging Radiometer Suite (VIIRS) satellite sensors. Level 3, mapped data were acquired at a spatial resolution of 4 km, and 8-day and monthly temporal resolutions from http://www.esa-oceancolour-cci.org, for the period January 1998 – December 2015. Satellite-derived Chl-a concentrations, before being spatially averaged over the NRS, were further evaluated and observable outliers were removed following a one-by-one visual inspection. We note that remotely sensed observations of Chl-a concentration in optically complex waters may be impacted by the presence of other optical constituents, such as coloured dissolved organic matter. However, comparisons with *in situ* data have demonstrated that remotely sensed datasets perform reasonably well in the Red Sea^61^, even in the optically complex coastal regions^29,36^. Thus, we are confident in the use of remotely sensed Chl-a data for studying interannual phytoplankton variability and phenology in the Red Sea. For further information, the reader is referred to previous literature regarding the OC-CCI product^28,62^ and its previous applications in the Red Sea and adjacent Arabian Sea^29,61,63,64^. In addition, we refer the reader to the OC-CCI Product User Guide at http://www.esa-oceancolour-cci.org/?q = webfm_send/318 for a more extensive overview of processing, sensor merging and uncertainty quantification.

### Estimation of phytoplankton phenological indices

The annual phenological indices of the seasonal phytoplankton bloom were estimated using the threshold criterion method^27,29,65^. The threshold criterion method is centred on the concept that the occurrence of a phytoplankton bloom corresponds to a significant increase in satellite-derived Chl-a above ‘normal’ concentrations^47^.

First, for the estimation of phytoplankton phenology indices, Chl-a data (8-day temporal resolution) from the original time series were isolated for the period spanning September 14^th^ 1997 – September 6^th^ 2015. Missing data due to the removal of outliers in the Chl-a time series were filled in using linear interpolation. The interpolation method used was based on the MATLAB subroutine *inpaint_nans*^66^, which interpolates missing data using a linear least squares approach^67^. It is worth mentioning that we tested an alternative method to fill in missing values using the 8-day climatological mean. No substantial differences were observed between the two methodologies, providing us with confidence in the use of our interpolation method. Visual inspection of the Chl-a seasonal cycle was then conducted for each year and a threshold criterion, defined as the median + 15% (computed based on the whole 18-year Chl-a time series), was selected. This threshold was found to be the most representative for capturing the initiation, duration and termination of the bloom for almost every year during the 18-year period (excluding 2010 when no clear phytoplankton bloom could be detected). We note that various thresholds have been utilised in different phenology studies [e.g.^27,29,68^] and the choice of threshold criterion is generally arbitrary and may depend on the type of analysis (e.g. interannual or climatological).

Next, Chl-a anomalies were computed by subtracting the threshold value from the annual time series and the cumulative sums of the anomalies were then produced. An increasing (decreasing) trend in the cumulative sums of anomalies represents periods when Chl-a concentrations rise above (below) the threshold value. The gradient of the cumulative sums of anomalies was then used to identify the transition points between increasing and decreasing trends. The timing of bloom initiation corresponded to the 8-day period when Chl-a concentrations first rose above the threshold criterion (i.e. when the derivative of the time series first changed sign). Similarly, bloom termination was computed as the time when the derivative first changed sign following the occurrence of the maximum Chl-a concentration in the time series (the bloom peak). Bloom duration corresponded to the number of 8-day periods between initiation and termination. The annual anomalies of the phenological indices were calculated by subtracting each index from the overall mean. The above technique was also applied on a pixel-by-pixel basis to reveal spatial patterns in phytoplankton phenology (see Fig. 5). In this case, pixels that may represent shallower, more optically complex coastal waters were removed from the analysis (coastal pixels were defined as <100 metres based on the GEBCO gridded bathymetry dataset). For convenience, 8-day periods are referred to as ‘weeks’ throughout the manuscript.

### SST data

A level 4, gap-free, blended SST dataset (GHRSST AVHRR_OI), downloaded from https://podaac.jpl.nasa.gov was used to investigate the relationship between Chl-a and SST in the NRS. This global SST product utilises data obtained from the Advanced Very High Resolution Radiometer (AVHRR) Pathfinder (V5) time series, combined with *in situ* ship and buoy observations. Data were acquired at a daily temporal resolution and mapped on a grid with a spatial resolution of 0.25° by 0.25°. Daily data were spatially averaged over the NRS and temporally averaged over 8-day periods to match the corresponding Chl-a remotely sensed dataset.

### Modelled data

Outputs acquired from the high resolution (~1.8 km) MIT general circulation ocean model (MITgcm), specifically designed to study the general circulation of the Red Sea^16,69^, were used to generate the vertical profiles of temperature in the NRS. The model covers the entire Red Sea and part of the Gulf of Aden, and was forced with reanalysis atmospheric data from the National Centers for Environmental Prediction (NCEP)^16,70^. It has successfully been used to describe the overturning circulation in the Red Sea and was further used for analysing the seasonal variability of the energetic mesoscale activity of the basin^35^. Estimations of the mean MLD, averaged across the whole NRS, were acquired using a temperature-difference based criteria. Commonly used values lie in the range of 0.01–1.0 °C for potential temperature^71^. For this study, a threshold value of 0.125 °C was chosen. To represent the overall temperature profile of the NRS, mean profiles were extracted and spatially averaged over the whole study area.

Modelled outputs of heat exchanges with the atmosphere were acquired from a high resolution (10 km), assimilated atmospheric product, developed at KAUST using the Advanced Research – Weather Research and Forecasting atmospheric model^72,73^. The model simulations were performed on a two-way nested domain (30 km and 10 km resolution) that covers the Red Sea and its adjacent regions. Initial and boundary conditions were acquired from the NCEP Final Analysis product^70^. Comparisons with *in situ* and other gridded data products have shown that this product successfully reproduces spatiotemporal patterns of wind, temperature and sea level pressure over the Red Sea^72,74^.

### Data analysis

Correlation analyses were used to statistically investigate relationships between datasets. Datasets were tested for normality using a one-sample Kolmogorov-Smirnov test. Following this, relationships between datasets were tested using either the Pearson Product Moment correlation or Spearman’s Rank correlation. All analyses of modelled and satellite datasets were conducted using the software package MATLAB (version R2015b, https://www.mathworks.com).

### Data availability

The datasets of air-sea heat flux and mixed layer depth analysed during the current study are available from the corresponding author on reasonable request. Bathymetry, ocean colour and sea surface temperature datasets are freely available at http://www.gebco.net, http://www.esa-oceancolour-cci.org and https://podaac.jpl.nasa.gov respectively.
